# Unilateral versus bilateral pedicle screw fixation in lumbar fusion: A systematic review of overlapping meta-analyses

**DOI:** 10.1371/journal.pone.0226848

**Published:** 2019-12-20

**Authors:** Yachao Zhao, Sidong Yang, Wenyuan Ding

**Affiliations:** 1 Department of Spine Surgery, The Third Hospital of Hebei Medical University, Shijiazhuang, China; 2 Hebei Provincial Key Laboratory of Orthopedic Biomechanics, The Third Hospital of Hebei Medical University, Shijiazhuang, China; Assiut University Faculty of Medicine, EGYPT

## Abstract

**Objectives:**

To carry out a systematic review on the basis of overlapping meta-analyses that compare unilateral with bilateral pedicle screw fixation (PSF) in lumbar fusion to identify which study represents the current best evidence, and to provide recommendations of treatment on this topic.

**Methods:**

A comprehensive literature search in PubMed, Embase, and the Cochrane Library databases was conducted to identify meta-analyses that compare unilateral with bilateral PSF in lumbar fusion. Only meta-analyses exclusively covering randomized controlled trials were included. Study quality was evaluated using the Oxford Levels of Evidence and Assessment of Multiple Systematic Reviews (AMSTAR) instrument. Then, the Jadad decision algorithm was applied to select the highest-quality study to represent the current best evidence.

**Results:**

A total of 9 studies with Level II of evidence fulfilled the eligibility criteria and were included. The scores of AMSTAR criteria for them varied from 5 to 9 (mean 7.78). The current best evidence detected no significant differences between unilateral and bilateral PSF for short-segment lumbar fusion in the functional scores, length of hospital stay, fusion rate, and complication rate. However, unilateral PSF involved a remarkable decrease in operative time and blood loss but increase of cage migration when compared with bilateral PSF.

**Conclusions:**

According to this systematic review, unilateral PSF is an effective method of fixation for short-segment lumbar fusion, has the advantages of reduced operative time and blood loss over bilateral PSF, but increases the risk of cage migration.

## Introduction

Lumbar fusion is an effective procedure commonly performed for treating lumbar degenerative disc diseases [1]. Generally, bilateral pedicle screw fixation (PSF) is deemed a standard instrumentation for lumbar fusion. However, the pronounced stiffness of bilateral PSF appears to cause undesired adverse effects such as reduced fusion rate, adjacent segment degeneration, and loss of bone mineral content [2,3]. In response to those concerns, unilateral PSF, which involves less rigidity, has been developed for lumbar fusion.

Biomechanical studies have demonstrated that unilateral PSF is able to maintain the initial stability after lumbar fusion, and decrease the influence of stress-shielding imposed on the fixed level and levels adjacent to the fusion [4,5]. In addition, numerous clinical studies have suggested that unilateral PSF is as effective as bilateral PSF for lumbar fusion but has the advantages of reduced operation time, blood loss, and implant cost [6–14]. A 5-year follow-up study by Toyone et al. [15] also found a lower occurrence of adjacent segment degeneration in patients undergoing unilateral PSF than that in patients who underwent bilateral PSF. Reversely, there exist studies indicating that unilateral PSF provided less stability than did bilateral PSF for lumbar fusion [16–20]. Due to its inherent asymmetry and reduced strength, unilateral PSF was reported to cause postoperative back pain, implant failure, more cage migration, and a relatively lower fusion rate when compared with bilateral PSF [8,21–23].

Recently, multiple meta-analyses have carried out a comparison of unilateral and bilateral PSF in lumbar fusion. However, those overlapping meta-analyses showed discordant results as well. Several studies suggested that unilateral and bilateral PSF were equally safe and effective for lumbar fusion [24–28]. However, the results of other studies indicated that unilateral PSF lead to more cage migration or a relatively lower fusion rate than bilateral PSF [29–33]. As a result, the above conflicting findings may bring uncertainty about which method of fixation is better for lumbar fusion.

The objectives of this study were to carry out a systematic review on the basis of overlapping meta-analyses regarding unilateral versus bilateral PSF in lumbar fusion to provide recommendations of treatment on this topic according to the current best evidence, and to identify potential limitations within current literature that require future research.

## Materials and methods

We carried out this systematic review according to the Preferred Reporting Items for Systematic Reviews and Meta-analysis (PRISMA) statement [34]. Ethical approval or patient consent is not required for this review.

### Literature search

A comprehensive literature search was carried out on July 27, 2019 using PubMed, Embase, and the Cochrane Library databases. The following keywords were adopted: unilateral, bilateral, lumbar, fusion, arthrodesis, systematic review, meta-analysis. Two reviewers independently performed the literature search. We first reviewed the titles and abstracts of all articles, and then obtained the full texts of articles that met the inclusion criteria. To identify other potentially eligible articles, the references were manually retrieved and screened as well. Disagreements were discussed and settled by consensus. Full electronic search strategy for PubMed: ((unilateral) OR bilateral) AND lumbar AND ((fusion) OR arthrodesis) AND ((systematic review) OR meta-analysis).

### Eligibility criteria

Inclusion criteria were listed as follow: (1) meta-analysis that compares unilateral and bilateral PSF in lumbar fusion; (2) inclusion of randomized controlled trials (RCTs) exclusively; (3) report of at least 1 outcome (functional scores, fusion rate, operation time, blood loss, complication rate, cage migration and so on); (4) English-language article. Correspondence, narrative reviews, annual meeting abstracts, systematic reviews without a meta-analysis or data pooling, and meta-analyses with no outcome data or including non-RCTs were excluded. In addition, the animal, cadaveric, and other non-clinical studies were also excluded.

### Data extraction

From each included study, the following data were extracted by two reviewers independently: first author, date of last literature search, publication journal and date, numbers of RCTs included, inclusion/exclusion criteria, restrictions to publication status and language, search databases, level of evidence, primary study design, software used for data analysis, whether sensitivity or subgroup analysis, Grading of Recommendations Assessment, Development, and Evaluation (GRADE) system, and publication bias were performed, conflict of interest, I^2^ statistic value of variables in each meta-analysis. In addition, clinical outcome data were also extracted, including the fusion rate, functional scores, operative time, length of hospital stay, blood loss, implant cost, nonunion rate, reoperation rate, total complication, general complication, infection, dura tear, implant-related complication, screw complication, and the cage migration. Disagreements were discussed and settled by consensus.

### Quality assessment

Methodological information of each included study was evaluated via the Oxford Levels of Evidence [35,36]. Furthermore, the Assessment of Multiple Systematic Reviews (AMSTAR) instrument was used to evaluate the study quality as well, which is a tool applied to methodological evaluation of meta-analyses and systematic reviews with good validity, reliability, and responsibility [37–39]. In this review, two reviewers evaluated the methodological quality of each included study independently. Disagreements were discussed and settled by consensus.

### Heterogeneity assessment

The heterogeneity across studies was evaluated using the I^2^ statistic which is a quantitative measurement to investigate inter-study variability. When I^2^ value > 50%, heterogeneity is deemed to exist between studies. If so, two reviews evaluated whether sensitivity or subgroup analysis was carried out to assess the robustness of data pooled and explore the potential causes of heterogeneity.

### Application of Jadad decision algorithm

The Jadad decision algorithm was applied to interpret discordant findings among meta-analyses. The possible causes of discordance, as mentioned by Jadad et al. [40], include different study question, inclusion/exclusion criteria, quality assessment, data pooling and extraction, and statistical analysis. Currently, this algorithm is widely used for providing treatment recommendations among conflicting meta-analyses on certain topics [41–45]. Two reviewers independently ran this algorithm, and then a consensus was reached as to which meta-analysis represents the current best evidence.

## Results

### Literature search

**Fig 1:**
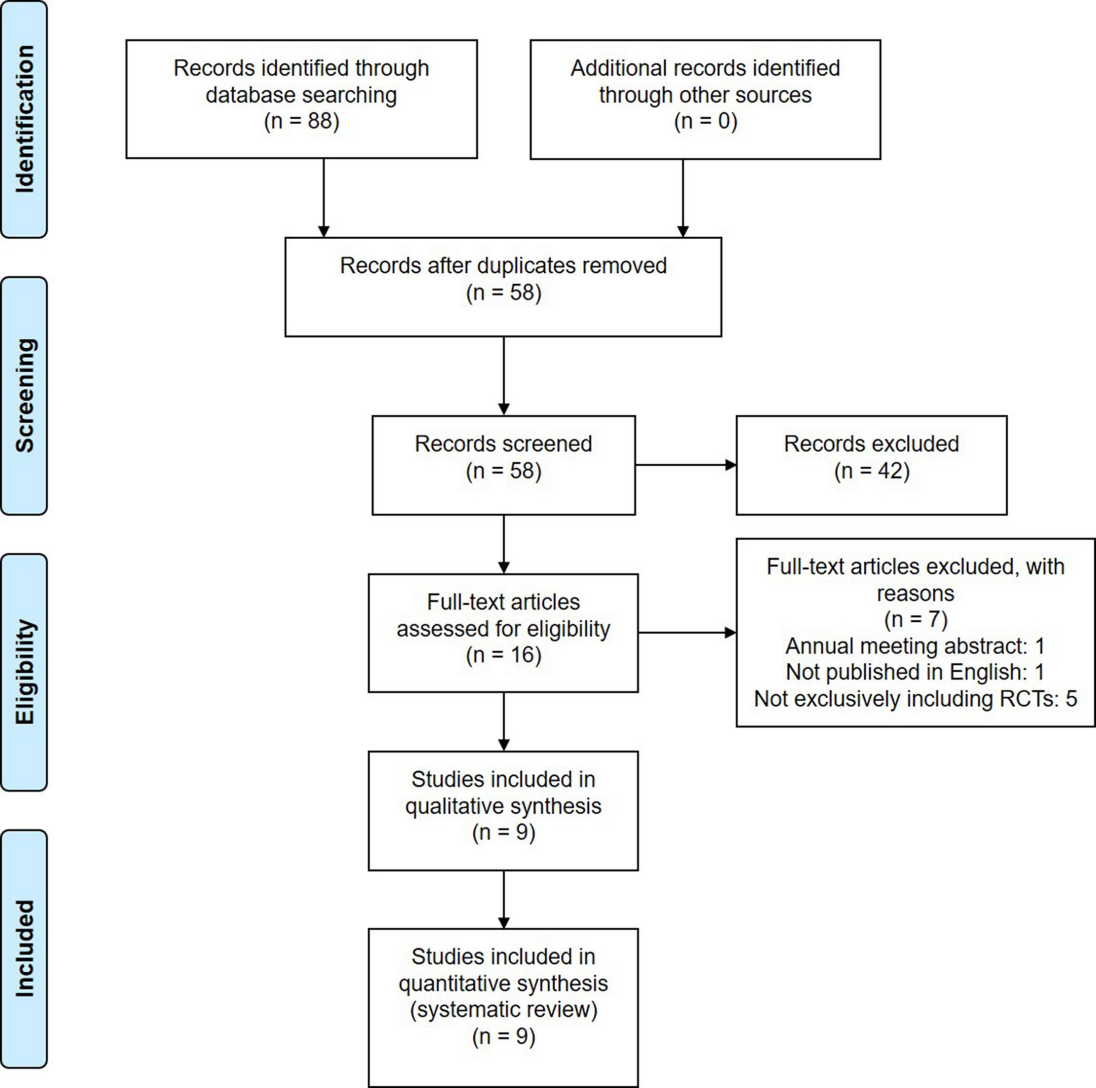
PRISMA flow diagram.

Fig 1 displayed the flowchart of study screening. The initial literature search identified 88 articles. After screening, 9 meta-analyses met the eligibility criteria and were finally included in this systematic review [27–29,33,46–50]. Those overlapping meta-analyses were published between 2014 and 2018 but in different journals, and the numbers of included RCTs among them ranged from 3 to 12 (Table 1). As shown in Table 2, the publication years of primary RCTs were between 2007 and 2015.

**Table 1:** Characteristics of each included study.

Author (Year)	Publication Date	Publication Journal	Last Literature Search Date	No. of RCTs Included
Hu XQ [50] (2014)	January, 2014	*PloS One*	August, 2013	7
Yuan C [29] (2014)	February, 2014	*Clinical Neurology and Neurosurgery*	NA	7
Wang L [49] (2014)	November, 2014	*BMC Surgery*	December, 2013	3
Molinari RW [48] (2015)	June, 2015	*Global Spine Journal*	September, 2014	10
Li X [47] (2015)	March, 2015	*Medical Science Monitor*	July, 2014	10
Xiao SW [46] (2015)	April, 2015	*European Spine Journal*	June, 2014	8
Ren S [27] (2016)	January, 2016	*International Journal of Clinical and Experimental Medicine*	April, 2016	12
Xin Z [28] (2016)	February, 2016	*International Orthopaedics*	April, 2015	11
Lu P [33] (2018)	November, 2018	*Journal of Orthopaedic Surgery and Research*	August, 2018	12

NA, not available; RCTs, randomized controlled trials.

**Table 2:** Primary trials included in each study.

Author (Year)	Fernández- Fairen (2007)	Feng (2011)	Lin (2012)	Aoki (2012)	Xie (2012)	Xue (2012)	Dahdaleh (2013)	Choi (2013)	Duncan (2013)	Lin (2013)	Shen (2013)	Zhang (2014)	Shen (2014)	Dong (2014)	Gu (2015)
Hu XQ [50] (2014)		+		+	+	+	+	+				+			
Yuan C [29] (2014)		+		+	+	+		+	+			+			
Wang L [49] (2014)							+	+			+				
Molinari RW [48] (2015)	+			+	+	+		+	+	+		+	+	+	
Li X [47] (2015)	+			+	+	+		+	+	+		+	+	+	
Xiao SW [46] (2015)				+	+	+		+		+		+	+	+	
Ren S [27] (2016)		+		+	+	+	+	+	+	+		+	+	+	+
Xin Z [28] (2016)	+	+	+	+	+	+	+	+	+			+		+	
Lu P [33] (2018)	+	+		+	+	+	+	+	+	+		+	+	+	

### Search methodology

Although the studies here conducted a comprehensive literature search, the search databases were discordant among them. All studies searched PubMed or Medline database. Of the 9 included studies, 7 searched Embase, 8 searched the Cochrane Library, whereas Web of Science, Ovid, Springer, CINAHL, Current Controlled Trials, National Guideline Clearinghouse, and other databases were retrieved in different studies. In addition, the restriction of publication language and status was discordant as well. Of the 9 studies, 4 only included articles published in English [29,46,47,50], 1 included both English- and Chinese-languages articles [28], 3 had no linguistic restriction [27,33,49], and the remaining 1 did not refer to restriction of publication language [48]. Only 1 study disclosed that unpublished data were reviewed [47], 1 study did not review unpublished data [50], and the remaining studies did not refer to restriction of publication status [27–29,33,46,48,49]. Details of search methodology applied by the included studies were presented in Table 3.

**Table 3:** Search methodology used by each study.

Author (Year)	Publication Language Restriction	Publication Status Restriction	PubMed	Medline	Embase	Cochrane Library	Web of Science	Ovid	Springer	CINAHL	Current Controlled Trials	National Guideline Clearinghouse	Other
Hu XQ [50] (2014)	Yes	Yes		+				+	+				
Yuan C [29] (2014)	Yes	NA		+	+	+							
Wang L [49] (2014)	No	NA		+	+	+	+						
Molinari RW [48] (2015)	NA	NA	+			+						+	
Li X [47] (2015)	Yes	No	+	+	+	+							+
Xiao SW [46] (2015)	Yes	NA	+	+	+	+							
Ren S [27] (2016)	No	NA		+	+	+							
Xin Z [28] (2016)	Yes	NA		+	+	+	+			+	+		+
Lu P [33] (2018)	No	NA	+		+	+							

NA, not available.

### Methodological quality

Based on the Oxford Levels of Evidence, we determined each study, which covers only RCTs, to be Level II of evidence (Table 4). Of the 9 included studies, 8 used RevMan for data analyses [27–29,46–50], only 1 used the Stata software [33]. In addition, 3 studies adopted the GRADE system [28,33,48], 7 studies conducted the sensitivity and/or subgroup analyses [27–29,33,46,48,50], and 4 studies assessed the possibility of publication bias [27,33,47,50]. As shown in Table 5, the scores of AMSTAR criteria for included studies varied from 5 to 9 (mean 7.78). Finally, the meta-analysis by Lu et al. [33], with an AMSTAR score of 9, was chosen as the highest-quality study.

**Table 4:** Methodological information of each study.

Author (Year)	Primary Study Design	Level of Evidence	Software Use	GRADE Use	Sensitivity or Subgroup Analysis	Publication Bias
Hu XQ [50] (2014)	RCT	Level II	RevMan	No	Yes	Yes
Yuan C [29] (2014)	RCT	Level II	RevMan	No	Yes	No
Wang L [49] (2014)	RCT	Level II	RevMan	No	No	No
Molinari RW [48] (2015)	RCT	Level II	RevMan	Yes	Yes	No
Li X [47] (2015)	RCT	Level II	RevMan	No	No	Yes
Xiao SW [46] (2015)	RCT	Level II	RevMan	No	Yes	No
Ren S [27] (2016)	RCT	Level II	RevMan	No	Yes	Yes
Xin Z [28] (2016)	RCT	Level II	RevMan	Yes	Yes	No
Lu P [33] (2018)	RCT	Level II	Stata	Yes	Yes	Yes

GRADE, Grading of Recommendations Assessment, Development, and Evaluation system; RCTs, randomized controlled trials.

**Table 5:** AMSTAR criteria for each study.

Items	Hu XQ [50] (2014)	Yuan C [29] (2014)	Wang L [49] (2014)	Molinari RW [48] (2015)	Li X [47] (2015)	Xiao SW [46] (2015)	Ren S [27] (2016)	Xin Z [28] (2016)	Lu P [33] (2018)
1. Was an a priori design provided?	0	0	0	0	0	0	0	0	0
2. Were there duplicate study selection and data extraction?	1	1	1	1	1	1	1	1	1
3. Was a comprehensive literature search performed?	1	0	1	1	1	1	1	1	1
4. Was the status of publication (i.e. grey literature) used as an inclusion criterion?	0	0	0	0	1	0	0	0	0
5. Was a list of studies (included and excluded) provided?	0	0	0	1	0	1	1	0	1
6. Were the characteristics of the included studies provided?	1	1	1	1	1	1	1	1	1
7. Was the scientific quality of the included studies assessed and documented?	1	1	1	1	1	1	1	1	1
8. Was the scientific quality of the included studies used appropriately in formulating conclusions?	1	1	1	1	1	1	1	1	1
9. Were the methods used to combine the findings of studies appropriate?	1	1	1	1	1	1	1	1	1
10. Was the likelihood of publication bias assessed?	1	0	0	0	1	0	1	0	1
11. Was the conflict of interest stated?	1	0	1	1	1	1	1	1	1
Total scores	8	5	7	8	9	8	9	7	9

### Heterogeneity assessment

All included studies assessed the heterogeneity using the I^2^ statistic (Table 6). No or slight heterogeneity was found in the fusion rate, nonunion rate, reoperation rate, total complication, general complication, infection, dura tear, implant-related complication, cage migration, and screw complication. Nevertheless, the heterogeneity of operation time, blood loss, and length of hospital stay was very large. There also existed different levels of heterogeneity in the functional outcomes. To further explore the potential sources of heterogeneity, 7 studies performed the sensitivity and/or subgroup analyses [27–29,33,46,48,50], as shown in Table 4.

**Table 6:** I^2^ statistic value of variables in each meta-analysis.

Items	Hu XQ [50] (2014)	Yuan C [29] (2014)	Wang L [49] (2014)	Molinari RW [48] (2015)	Li X [47] (2015)	Xiao SW [46] (2015)	Ren S [27] (2016)	Xin Z [28] (2016)	Lu P [33] (2018)
VAS					63%	47%			21.1%
VAS for leg pain	70%			89%			64%		
VAS for back pain	50%		0%	74%			32%	0%	
JOA	59%			77%	54%		59%		48.2%
ODI	34%		0%	51%	0%	20%	0%	50%	3%
SF-36									37.9%
SF-36 Mental health						0%	0%		59.6%
SF-36 General health						0%	0%		28.4%
SF-36 Physical function						0%	0%		4.5%
Operative time	95%				97%	98%	94%	97%	98.1%
Blood loss	96%		57%		98%	99%	96%	99%	98.7%
Length of hospital stay	97%				99%		94%	99%	99.5%
Implant cost	NA								
Fusion rate		0%	0%		0%	0%	0%	7%	0%
Total complication	0%	11%	0%				0%		0%
General complication				0%		0%			
Infection				0%			0%		
Dura tear							0%		
Nonunion rate	0%			0%					
Reoperation rate	0%			5%			0%		
Device-related complication								7%	
Implant-related complication						0%			
Cage migration		0%		57%		NA			0%
Screw complication					0%		0%		
Screw failure				0%					

NA, not available; VAS, Visual Analog Scale; JOA, Japanese Orthopedic Association; ODI, Oswestry Disability Index; SF-36, Short-Form Health Survey.

### Results of Jadad decision algorithm

**Fig 2:**
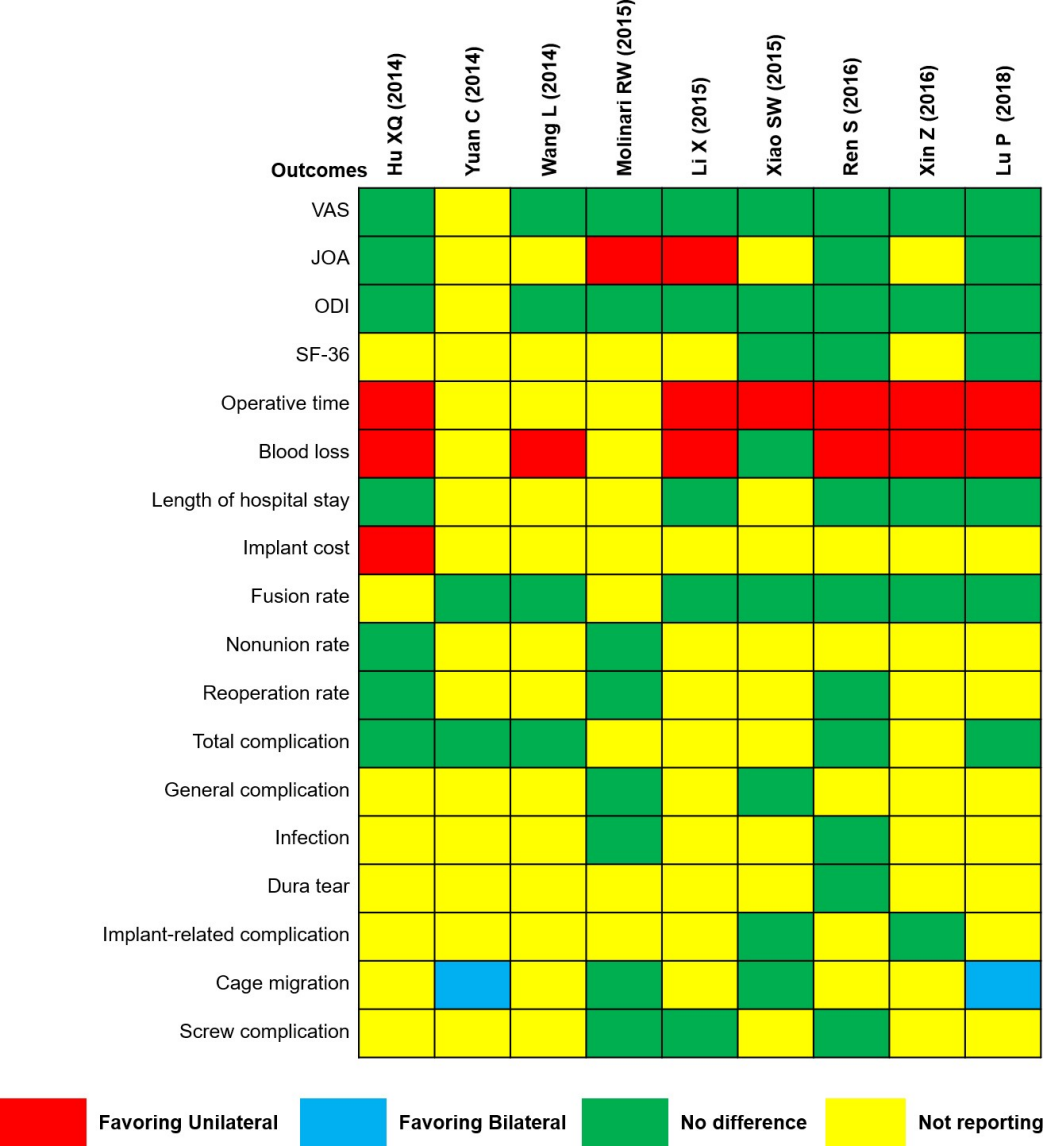
The pooled results of each included meta-analysis. VAS, Visual Analog Scale; JOA, Japanese Orthopedic Association; ODI, Oswestry Disability Index; SF-36, Short-Form Health Survey.

**Fig 3:**
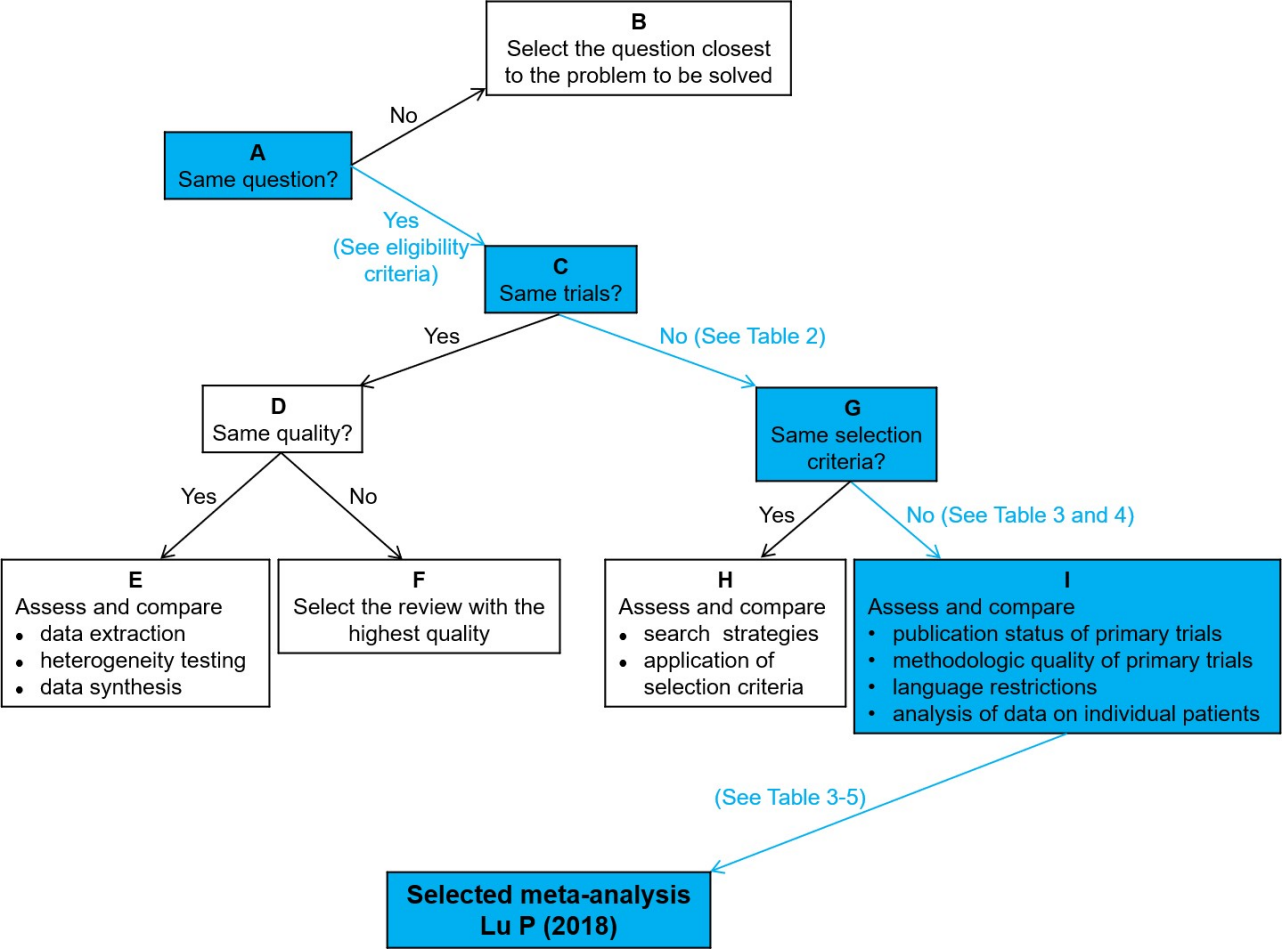
The flowchart of Jadad decision algorithm.

Fig 2 showed the pooled results of each meta-analysis. The Jadad decision algorithm was adopted to identify which study represents the current best evidence to provide treatment recommendations. Considering that the 9 included meta-analyses focused on the same clinical question while they did not cover the same primary trials and criteria of study selection, the highest-quality study can be selected based on the methodological quality and publication status of primary trials, language restrictions, and data analysis on individual patients (Fig 3). Eventually, the meta-analysis by Lu et al. [33] was identified as the highest-quality study with more RCTs using the Jadad decision algorithm. This study detected no significant differences between unilateral and bilateral PSF for lumbar fusion in the functional scores, length of hospital stay, fusion rate, and complication rate. However, unilateral PSF significantly reduced the operative time and blood loss but increased cage migration when compared with bilateral PSF.

## Discussion

Currently, numerous RCTs have made a comparison of unilateral and bilateral PSF in lumbar fusion, whereas their findings are conflicting as to which method of fixation is better [6–13,21,23,51–54]. To further clarify this issue, multiple meta-analyses based on RCTs, which provide the highest level of evidence, have also compared the two fixation methods for lumbar fusion [27–29,33,46–50]. Nevertheless, the results are discordant as well. Recently, several systematic reviews have been conducted based on overlapping meta-analyses [41–45], which can provide information that may assist decision makers in choosing the highest-quality study among meta-analyses with discordant findings.

The objectives of this study were to carry out a systematic review on the basis of overlapping meta-analyses based on RCTs regarding unilateral versus bilateral PSF in lumbar fusion to identify which study represents the highest level of evidence to provide treatment recommendations on this topic. After a comprehensive literature search, 9 meta-analyses were finally included in this review [27–29,33,46–50]. Jadad et al. [40] designed a decision tool to allow selection of the highest-quality study from among conflicting meta-analyses, which has been widely used in medical fields [41–45]. Finally, the meta-analysis by Lu et al. [33], which included the most RCTs with Level II of evidence, was chosen as the highest-quality study based on the Jadad decision algorithm. The current best evidence detected no obvious differences between the two fixation methods for lumbar fusion in functional scores, length of hospital stay, fusion rate, and complication rate. Furthermore, unilateral PSF was superior to bilateral PSF in operation time and blood loss, but increased cage migration. Based on the above findings, Lu et al. [33] concluded that unilateral PSF is recommended as the optimal fixation method for lumbar fusion.

Interestingly, the 9 included studies were published within the last few years and focused on the same study question, whereas they did not cover the same primary RCTs and criteria of study selection [27–29,33,46–50]. As a result, findings were discordant regarding unilateral versus bilateral PSF in lumbar fusion. According to this review, no significant differences were detected between the two methods in VAS, ODI, SF-36 scores, and fusion rate. As for JOA scores, 3 studies found no significant differences between the two methods [27,33,50], but 2 studies reported data in favor of unilateral PSF [47,48]. In conclusion, the current evidence suggested that both unilateral and bilateral PSF were effective fixation methods for lumbar fusion though various levels of heterogeneity were reported in the functional scores. The reason may be that the effectiveness mainly depends on sufficient decompression and fusion rather than fixation method, type of operation, or surgical segments which are the potential sources of heterogeneity between studies.

With regard to surgical trauma, 6 studies showed that unilateral PSF significantly reduced the operative time and/or blood loss in comparison with bilateral PSF for lumbar fusion [27,28,33,47,49,50]. While no remarkable difference was detected in blood loss, the study by Xiao et al. [46] also reported data favoring unilateral PSF. In addition, unilateral PSF achieved a similar hospital stay with bilateral PSF, despite the fact that lesser dissection of soft tissues benefits functional recovery. Although there was large heterogeneity across studies, which was associated with type of operation, surgical segments, or skill of the surgeons, the current evidence concluded that unilateral PSF was superior to bilateral PSF in operation time and blood loss due to its not dissecting the soft tissues on the contralateral side.

Despite unilateral PSF was reported to cause adverse effects due to its inherent asymmetry and reduced stability [8,21–23], no significant differences were detected between them for lumbar fusion in the fusion rate, nonunion rate, reoperation rate, general complication, and total complication according to this review. With respect to implant-related complications, 5 studies found no significant differences between the two methods [27,28,46–48]. Of the 4 studies that reported data of cage migration, 2 detected no significant differences but an increased incidence in patients undergoing unilateral PSF [46,48], the other 2, including the highest-quality study, favored bilateral PSF [29,33]. Risk factors for cage migration are multiple such as cage type, cage position, cage material, cage size, disc space shape, multilevel fusion, degenerative scoliosis, and unilateral PSF [55–59]. The increased cage migration in patients who underwent unilateral PSF may be caused by inherent asymmetry that results in asymmetry. No or slight heterogeneity exists between studies in the above outcomes. To sum up, the current evidence demonstrated that unilateral PSF increased the risk of cage migration in lumbar fusion when compared with bilateral PSF.

This study had several limitations. First, only articles that published in English were included. Non-English articles meeting the eligibility criteria may have been excluded. Second, meta-analysis that covers only RCTs was identified as Level II of evidence. Therefore, we cannot provide treatment recommendations on this topic based on Level I of evidence. Third, none of the primary trials had data for more than 5 years. Long-term follow up is required to further verify these findings. Fourth, the current literature mainly focused on comparing unilateral and bilateral PSF in short-segment lumbar fusion. A comparison of them in long-segment lumbar fusion could not be made. Last but not least, this review may be underpowered by other potential limitations and bias within the included meta-analyses and primary trials.

## Conclusions

This is the first systematic review on the basis of overlapping meta-analyses to analyze unilateral versus bilateral PSF in lumbar fusion. According to the current best evidence, no significant differences were detected between unilateral and bilateral PSF for short-segment lumbar fusion in functional scores, length of hospital stay, fusion rate, and complication rate. However, unilateral PSF involved a remarkable decrease in operative time and blood loss but increase of cage migration when compared with bilateral PSF. In conclusion, unilateral PSF is an effective method of fixation for short-segment lumbar fusion, has the advantages of reduced operative time and blood loss over bilateral PSF, but increases the risk of cage migration.

## Supporting information

S1 File PRISMA checklist. (DOC)
